# Investigation of Microwave Absorption Performance of CoFe_2_O_4_/NiFe_2_O_4_/Carbon Fiber Composite Coated with Polypyrrole in X-Band Frequency

**DOI:** 10.3390/mi11090809

**Published:** 2020-08-26

**Authors:** Rozhin Sadeghi, Abbas Sharifi, Marta Orlowska, Isabelle Huynen

**Affiliations:** 1 Ceramic Department, Materials and Energy Research Center, Alborz 31787-316, Iran; 2 Department of Chemistry, Arak University, Arak 38156-879, Iran; sharifiabbas@yahoo.com; 3 Faculty of Materials Science and Engineering, Warsaw University of Technology Woloska 141, 02-507 Warsaw, Poland; marta.orlowska.dokt@pw.edu.pl; 4 ICTEAM Institute, Université Catholique de Louvain, 3 Place du Levant, 1348 Louvain-la-Neuve, Belgium

**Keywords:** CoFe_2_O_4_/NiFe2O_4_, carbon fiber, polypyrrole, electromagnetic properties, microwave absorber

## Abstract

The current research reports the preparation of a microwave absorber containing CoFe_2_O_4_/NiFe_2_O_4_/Carbon fiber (H/S/CF) coated with polypyrrole polymer (PPy@H/S/CF) through sol-gel and in-situ polymerization processes. X-ray diffraction (XRD), field emission scanning electron microscopy (FESEM), vibrating sample magnetometer (VSM), and a vector network analyzer (VNA) are utilized to evaluate the features of the prepared composite. The microstructure analysis results revealed carbon fibers well decorated with submicron-size particles having hard/soft magnetic phases and thoroughly coated with polymer. The paraffin-based microwave absorber sample filled with 45 wt.% of PPy@H/S/CF has simultaneously both magnetic and dielectric losses in the 8.2–12.4  GHz frequency range. The absorber is used in a Salisbury screen configuration aiming at reducing the radar cross-section of objects. A minimum reflection loss of −55  dB at 10.6 GHz frequency with 5 GHz bandwidth is obtained for the sample with a 2  mm thickness. Different mechanisms, such as interfacial polarization, ferromagnetic resonance, and electron hopping, are the main factors for achieving such an appropriate microwave absorption. These results suggest that the PPy@H/S/CF composite is an ideal candidate for microwave absorption applications requiring high performance and low thickness.

## 1. Introduction

With the ever-growing increase of communications systems, there is a crucial need for compact lightweight devices combining compactness and reliability. The constraints are antagonizing if we consider that reduced size increases the risk of harmful electromagnetic interferences between electronic components. The same risk occurs from the wireless propagation which may be detrimental to both electronic devices and human body [1]. Therefore, for more than one decade, research has been conducted on efficient microwave absorbing structures (MAS) aiming at blocking such interferences. In particular, MAS can be designed to reduce the radar cross-section (RCS) of various objects for military as well civil applications: an absorbing shield covering the target is used to reduce by absorption the signal reflected by the target towards the radar, making the target almost invisible to radar (concept of stealth). Well-known applications of this are military airplanes (stealth bombers) and towers of wind farms, that need to be stealth for security reasons, i.e., to ensure invisibility or avoid spurious signatures perturbing the control of the traffic in civil air traffic. A dedicated family of absorbers is developed to reduce the RCS: the Salisbury screen configuration [2,3], based on an absorbing layer covering the target is assumed to be metallic, is thus reflective. The conductivity of the layer and its thickness can be tuned in order to match the frequency and bandwidth of the absorption, depending on the applications. A well-known case is radar communications at X band (8–12.4 GHz) [4].

Polymer composites combining conductive charges and polymer hosting matrix offer several advantages; high conductivity at low loading rate owing to very good dispersion, mechanical stiffness, as well as the possibility of conformability for stealth application, lightweight as compared to metallic shields, etc. Various MAS were studied [5,6], where carbon fillers, including carbon black, incorporated at different concentration levels in single as well as multilayer configurations, induce reflection below −10 dB. A good absorber aims at minimizing the reflection of a signal incident to its surface, while maximizing the absorption through its thickness. Conductive composites are suited to attenuate the signal, however conductive charges may also increase the reflection as do metallic shields. An adequate strategy in order to counterbalance this effect consists in incorporating magnetic charges into the composite [7,8,9,10]. This combination allows to decrease the equivalent impedance at input interface as close as possible to that of air. The reflection is consequently reduced, and absorption favored via the penetration of the signal in the absorber.

Our paper exploits this strategy. Indeed, we propose a microwave absorber in Salisbury screen configuration where the conductive layer is based on composite materials combining carbon fibers (CF) and CoFe_2_O_4_/NiFe_2_O_4_ magnetic particles (MNP), covered by polypyrrole (PPy). Polypyrrole has already demonstrated its efficiency for EMI absorption [11,12,13,14]. Section 2 details the synthesis procedure of CF and MNP, as well as morphological and microwave characterization tools. Section 3 provides the structural/morphological analysis as well as the demonstration of the absorption performances of our composite through measurements of electromagnetic parameters and resulted minimal reflection losses observed in our structures. It also provides a comparison of performances of our absorber with literature, while Section 4 concludes our work.

## 2. Materials and Methods

### 2.1. Preparation of CoFe_2_O_4_/NiFe_2_O_4_/Carbon Fiber Composite

In this study, carbon fibers with a diameter of 7–9 μm were used. Prior to the surface treatment, the carbon fibers were cut 1–2 mm in length. The carbon fibers were ultrasonic washed with acetone for 1 h and then washed by distilled water. Then the carbon fibers were treated with nitric acid 65% for 3 h and finally washed by distilled water. Finally, it is dried at 70 °C for 5 h, the final obtained coated powder is denoted as PPy@H/S/CF.

Hard/soft magnetic composite powder with the nominal composition of CoFe_2_O_4_/NiFe_2_O_4_ with 1:1 mass ratio was synthesized by sol–gel method. The aqueous solutions of Fe(NO_3_)_3_ H_2_O, Ni(NO_3_)_2_ 6H_2_O and Fe(NO_3_)_3_ 9H_2_O, Co(NO_3_)_2_ 6H_2_O were separately prepared by dissolving into distilled water and magnetically stirred at 70 °C. Then these two solutions were mixed together and appropriate amount of CF (30 wt.% of procedure weight) was added in a solution and stirred for 1 h.

The pH value of the obtained solution is adjusted to 7 by adding drop-wisely NaOH solution (2 mol). After that, the mixture solution was dried at 200 °C for 4 h, the black carbonaceous precursor powder was obtained. Finally, the obtained powder was sintered at 600 °C for 1 h in Ar atmosphere, and the final obtained powder is denoted as H/S/CF.

### 2.2. Coating Composite Powder with Polypyrrole

To perform the coating of polypyrrole (PPy) on the surface of H/S/CF composite powder, 2 cc of pyrrole and 1 g of the mixture are added into a 50 mL of 0.5 mol HCl solution (Solution A). The solution is mechanically stirred in an ice bath. Then, an appropriate amount of APS is dissolved in 50 cc of 0.5 M HCl solution (Solution B). Finally, solution B is added dropwise into the solution A. The polymerization process is allowed to proceed for 2 h at 0–5 °C. The obtained powder is washed several times with distilled water. Finally, it is dried at 70 °C for 5 h, and the final obtained coated powder is denoted as PPy@H/S/CF.

**Figure 1:**
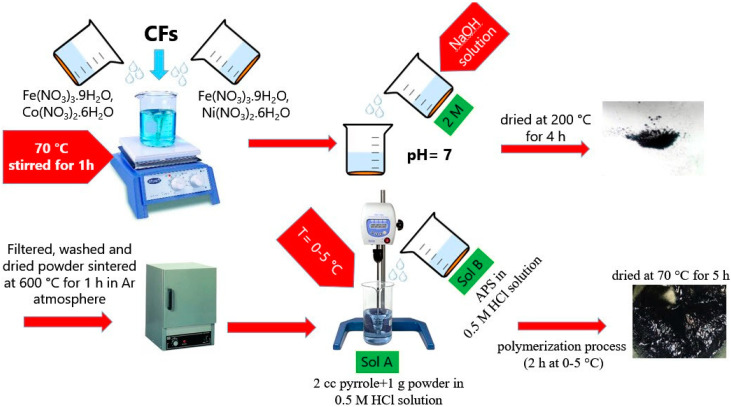
Schematic illustration of the synthesis process.

The whole procedure is illustrated in Figure 1.

### 2.3. Preparation of X Band Absorber Samples

45 wt.% of each composite (H/S/CF and PPy@H/S/CF) were separately mixed in molten paraffin and homogenized well with ultrasonic vibration for 20 min. Finally, samples were molded in the dimension of 22.86 × 10.16 × 2 mm in order to match the dimensions of the rectangular section of a standard waveguide operating at X band. Considered absorbers are thus in solid phase.

### 2.4. Evaluation of Properties

The powder X-ray diffraction (XRD, XMD 300, Cu-Kα, Unisantis, Georgesmarienhutte, Germany) technique is used for determining the crystal structures of the products. The morphology of the prepared powders was taken using a TESCAN- MIRA III field emission scanning electron microscope (TESCAN, Brno, Czech Republic). The magnetic properties of the samples are measured with a vibrating sample magnetometer (VSM) at room temperature up to 1.5 T. Microwave absorption characteristics (*ε*, *μ*, reflection loss (*RL*)) in the range of X band frequency (8.2–12.4 GHz) are measured using vector network analyzer (VNA, Agilent 8510C, Agilent, Santa Clara, CA, United States) by a waveguide method.

The S parameters (S_11_/S_22_ and S_21_/S_12_) were measured by VNA in X band frequency via waveguide method (waveguide width = 15.77 mm, holder length = 7.89 mm, distance from port 1 = 2.5 mm). Each sample having dimensions 22.86 × 10.16 × 2 mm. is inserted in a rectangular waveguide section in order to fully fill its section, so that the signal travels inside the waveguide through the 2 mm-thick sample in order to probe the material under scope (molded paraffin containing composite). For the characterization of the return losses associated to the absorption capability discussed in Section 3.5, each sample is backed by a metallic plate in order to characterize the sample in reflective configuration. The complex permittivity and permeability were evaluated through transmission and reflection measurements [14]. The electromagnetic parameters were deduced from the measured S parameters. The whole procedure is described in [15].

## 3. Results and Discussion

### 3.1. Phase Identification Analysis

**Figure 2:**
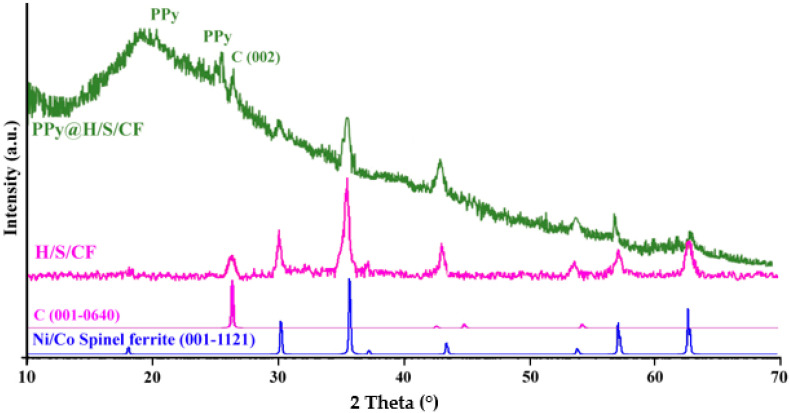
X-ray diffraction patterns of H/S/CF and polypyrrole polymer (PPy@H/S/CF) samples.

Figure 2 depicts the X-ray diffraction analysis patterns of H/S/CF and PPy@H/S/CF samples. The X-ray diffraction pattern of the H/S/CF product exhibits eight characteristic peaks of face centered cubic (FCC) system of cobalt spinel ferrite (CoFe_2_O_4_, reference code: 001-1121) and nickel spinel ferrite (NiFe_2_O_4_, reference code: 010-325) at 18.12, 30.27, 35.74, 37.28, 43.47, 53.88, 57.16, and 62.72° related to Miller indices of (111), (220), (311), (222), (400), (422), (511), and (440), respectively, which are overlapped at the same angles. The result suggests a simultaneously formation of magnetic CoFe_2_O_4_ and NiFe_2_O_4_ phases in the H/S/CF sample. Moreover, an obvious characteristic peak at 25.38° is related to the (002) plane of the graphite structure of carbon fiber which demonstrates the stability of CFs structure during synthesize process.

As it can be seen in PPy@H/S/CF composite diffraction pattern, all characteristic peaks are weakened due to the completely coated particles with PPy polymer with low degree of crystallinity. Specifically, a broad diffraction peak in the range of 21–25° indexed as PPy polymer is caused by polymer chains scattering at the interplanar spacing of polypyrrole polymer.

### 3.2. Field Emission Scanning Electron Microscopy (FESEM) Morphology

**Figure 3:**
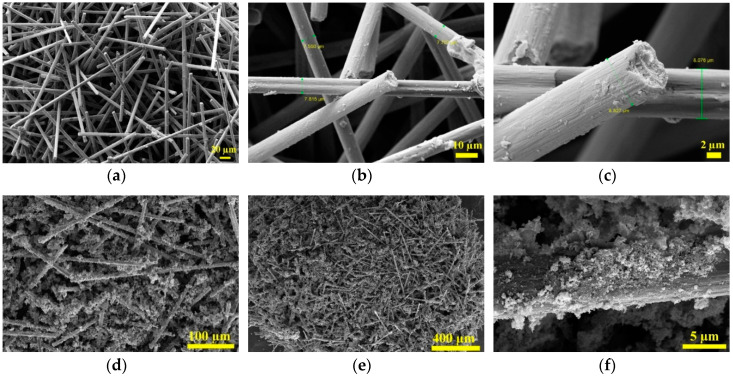
Field emission scanning electron microscopy (FESEM) images of (**a**–**c**) treated carbon fiber and (**d**–**f**) CoFe_2_O_4_/NiFe_2_O_4_/Carbon fiber (H/S/CF).

Field emission scanning electron microscopy (FESEM) images of carbon fibers is demonstrated in Figure 3a–c, which shows that the diameter of the CFs is about 7–9 µm with approximately uniform dimensions. Also, it shows a relatively rough surface because of the surface oxidation treatment. Figure 3d–f exhibits the microstructure of H/S/CF sample. According to the images, CFs are surrounded and decorated well with magnetic particles while H/S particles are almost tightly agglomerated on the surface of carbon fiber.

**Figure 4:**
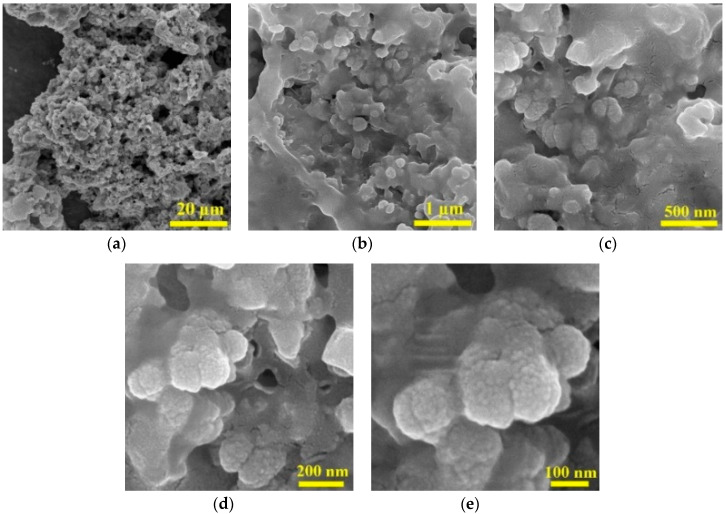
FESEM images of PPy@H/S/CF at different magnification. (**a**) scale bar: 20 μm; (**b**) scale bar: 1 μm; (**c**) scale bar: 500 nm; (**d**) scale bar: 200 nm; (**e**) scale bar: 100 nm.

FESEM images of the as-prepared PPy@H/S/CF composite are illustrated in Figure 4a–e. It can be clearly seen that polypyrrole polymer coated and adhered well on the surface of the particles having submicron size. Due to the appropriate dosage of pyrrole monomer to powder (2:1), leading to high adhesion of the polypyrrole on the surface of particles, we couldn’t find any separated particles without coating. Polypyrrole coating on the particles surface forming almost core-shell like structure will produce many interfaces between particles, which resulted in scattering and reflecting of the waves, as well as increasing interfacial polarization.

**Figure 5:**
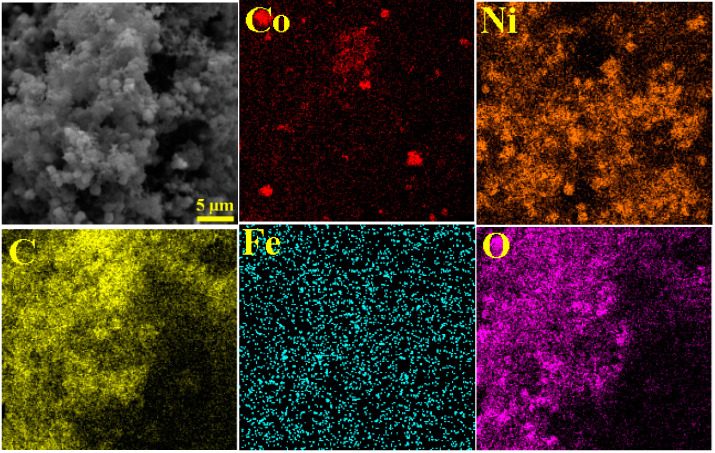
Elemental mapping analysis of Co, Ni, C, Fe, O for PPy@H/S/CF sample.

In order to better discern the composition of the sample, elemental mapping analysis has been done. Figure 5 illustrates the Energy-dispersive X-ray spectroscopy (EDX) mapping analysis of Co, Ni, C, Fe, and O for the PPy@H/S/CF sample. The results confirm the presence of all these elements in the sample which distributed quasi uniformly in the studied section. This result demonstrates the successful preparation of the powder sample.

### 3.3. Magnetic Properties

**Figure 6:**
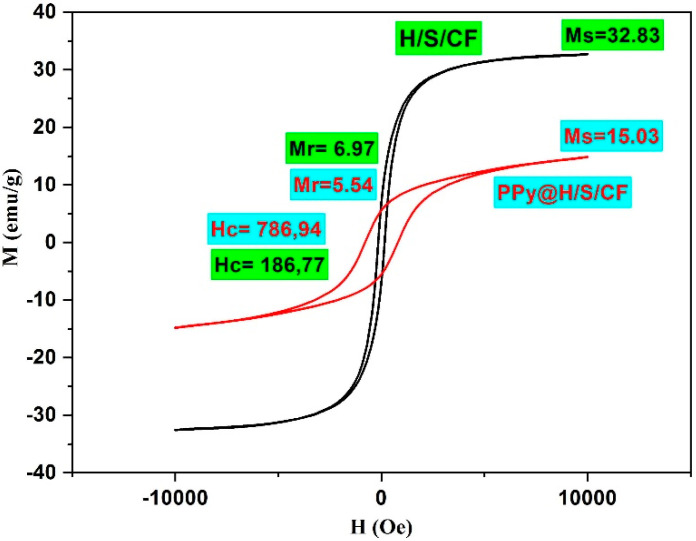
Hysteresis loops of H/S/CF and PPy@H/S/CF samples.

Magnetic features of as-prepared samples were examined via vibrating sample magnetometer (VSM) at room temperature. Figure 6 depicts the S-like shape hysteresis loops of H/S/CF and PPy@H/S/CF samples. According to the results, H/S/CF sample showed the maximum value of saturation magnetization (*M_s_*) (32.83 emu/g), whilst, this parameter value has diminished to 15.03 emu/g for PPy@H/S/CF sample. According to *M_s_* = *φ·m_s_* equation, the volume of particles (*φ*) and a single particle’s saturation moment (*m_s_*) are two main parameters which are entirely linked to M_s_. The presence of non-magnetic polymer on the surface of particles diminish the saturation magnetization (*M_s_*) and remanent magnetization (*M_r_*) of composite. It is seen from the hysteresis loop of H/S/CF sample that combination of two hard/soft magnetic powders demonstrates single-phase magnetic properties which suggests well exchange coupling phenomena. The increment in coercivity parameter after coating particles with polymer may be linked to surface anisotropy and also interaction between particles. The larger magneto-crystalline anisotropy energy causes the higher HC value, which improves the microwave absorption capability in the prepared sample.

### 3.4. Electromagnetic Parameters

**Figure 7:**
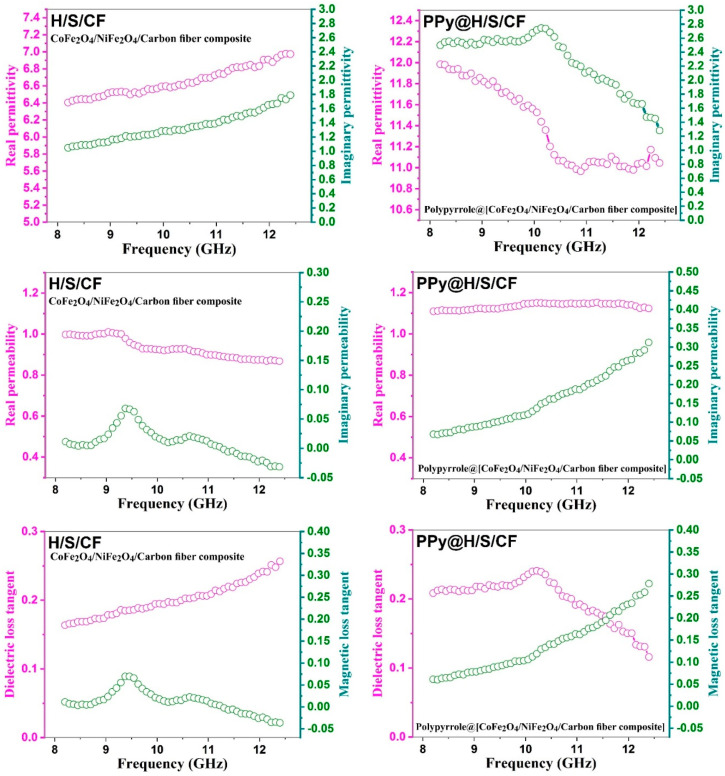
Electromagnetic properties of composite samples. Left: H/S/CF, right: PPy/H/S/CF. Row 1: permittivity *ε*, row 2: magnetic permeability *μ*, row 3: corresponding loss tangent factors tan *d_ε_* and tan *d_μ_*.

Figure 7 shows the relative dielectric permittivity *ε* and relative magnetic permeability *μ*, as well as their corresponding loss tangent factors tan *δ_ε_* and tan *δ_μ_* for the samples H/S/CF (left) and PPy@H/S/CF (right). Clearly the latter sample exhibits higher values of permittivity, permeability, and losses. This is obviously ascribed to the presence of PPy which induces higher magnetic losses associated to coercity and higher dielectric losses due to its conductive nature [16]. Indeed, tan *δ* = *σ*/(2*πf ε*′ *εo*). PPy is an organic conductive polymer. The presence of conducting polymer on the surface of the particles may increase interfacial polarization and obviously increase the reflection loss of the composite. We have tested this type of polymers previously, and by increasing the weight fraction of the polymer to powder the interfacial polarization increase and the reflection loss increase accordingly [17,18,19,20]. Other works also report the use of polypyrrole for microwave absorbers [21,22,23,24,25,26,27,28].

**Figure 8:**
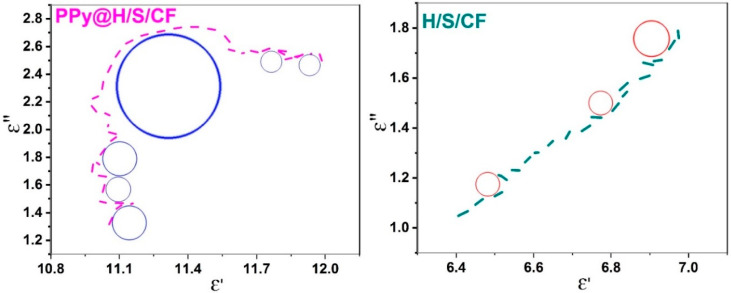
Cole-Cole representation of permittivity (*ε*″ versus *ε*′) for sample PPy@H/S/CF (**left**) and H/S/CF (**right**).

Another representation of electromagnetic parameter *ε* is provided at Figure 8: The Cole-Cole plot of *ε*″ versus *ε*′. Sample H/S/CF exhibits three circles associated to relaxation frequencies, while sample PPy@H/S/CF has five. The presence of the PPy coating favors more interfacial interactions between CF, MNP and PPy inducing relaxation. PPy also induces higher values of imaginary part *ε*″ due to its conductive nature [16].

### 3.5. Microwave Absorption Capability

**Figure 9:**
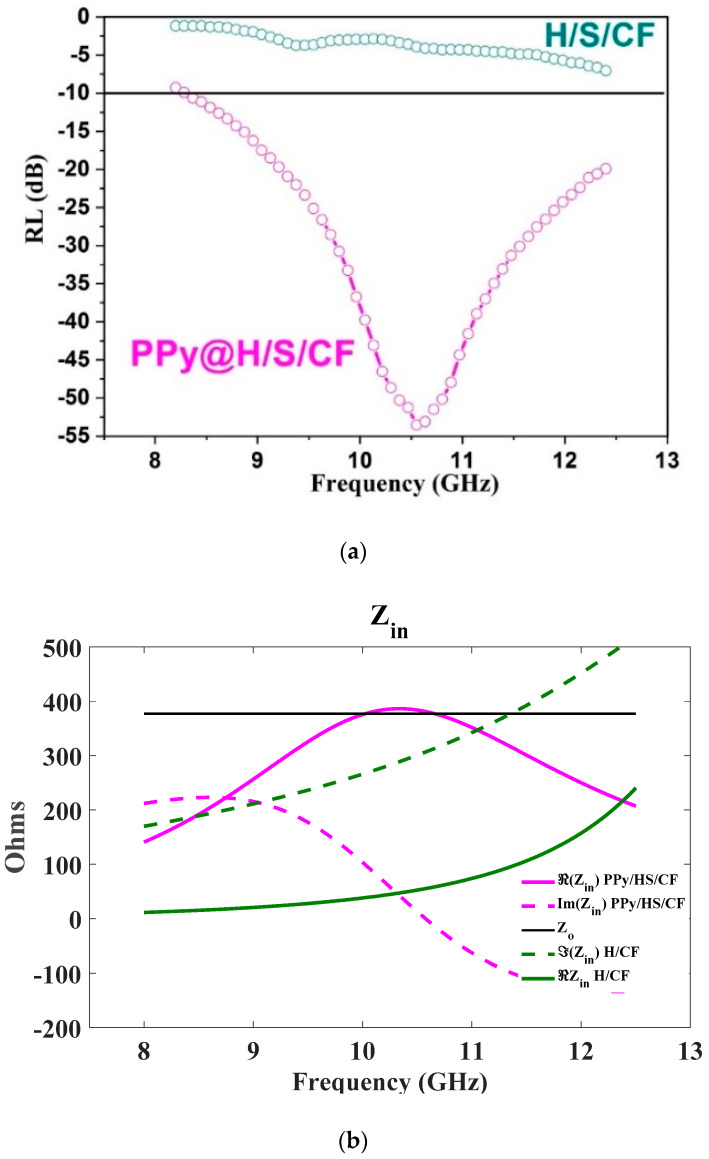
(**a**) Return losses (*RL)* according to Equation (1) for sample H/S/CF and PPy@H/S/CF. (**b**) Frequency dependence of complex impedance *Z_in_* for H/S/CF and PPy/H/S/CF.

The microwave absorption capability of our samples was also studied via the evaluation of *RL* associated to reflection of samples in a Salisbury screen configuration, that is when the absorbing sample is backed by a metallic plate. This configuration mimics the radar operation where signal incident to a target is reflected, allowing its detection by the radar. For stealth applications, *RL* has to be minimized in order to make the target invisible. The Salisbury configuration is adequate to evaluate the absorption capability of a material through the measurement of *RL* since if *RL* is minimized, it means that all signal has been absorbed in the sample so that almost nothing is reflected (nor transmitted since the metallic plate blocks the propagation. *RL* in Salisbury configuration expresses as the reflection coefficient at input interface of an absorber and depends on the input impedance *Z_in_* [29]:

(1) $$\mathrm{RL}=20{\;\log}_{10}\;\left(\frac{Z_{\mathrm{in}}-Z_{O}}{Z_{\mathrm{in}}+{\;Z}_{O}}\right)$$

(2) $$Z_{in}=\;Z_{o\;}\sqrt{\frac{\mu }{\varepsilon }}\;tan\;h\left(\gamma \;d\;\right)$$

(3) $$\gamma =\;j\;2\;\pi \;f\;\sqrt{\;\varepsilon \;\left(\;1-\tan\;\delta_{\varepsilon }\right)\;\mu \;\left(1-j\;tan\;\delta_{\mu }\right)}/c_{O}$$

where *Z_o_* is the free-space impedance equal to 377 Ohms, *f* is the frequency, *d* is the thickness of the sample, *c_o_* is the light velocity in air, while *ε_r_*, *μ_r_*, tan *δ_ε_*, and tan *δ_μ_* are permittivity, permeability, and associated loss tangent factors of each sample measured at Figure 7. The electromagnetic parameters of the sample have thus an impact on *RL*. Figure 9a compares the *RL* values versus frequency at X band for our two samples. H/S/CF sample fails to meet the *RL* < −10 dB criterion qualifying a good absorber, while sample PPy@H/S/CF succeeds over the whole frequency range. Moreover, it achieves a minimal value of nearly −55 d B at 10.6 GHz and corresponding −10 dB bandwidth (*BW*) is roughly 5 GHz. This value competes very well with the values *RL* = −32.9 dB and *BW* = 4.8 GHz reported in [30] for Cobalt PPy composite, and *RL* = −25 dB with *BW*= 3 dB reported in [14] for PPy/Ni_0.25_Co_0.25_Ti_0.5_Fe_2_O_4_/SrCoTiFe_10_O_19_/Cu composite.

The absorber operating in Salisbury configuration exploits a resonant behavior occurring when the thickness *d* of the sample is a multiple of the operating wavelength; at the corresponding frequency the reflected signal returns to the input of the absorber with a 180° phase shift while being almost totally attenuated by absorption induced by the lossy material, so that *Z_in_* becomes a real number proportional to the losses in the material. The higher the losses in the material, the more *Z_in_* is close to *Z_o_*, minimizing thus *RL* by virtue of Equation (1). Figure 9b shows the behavior of *Z_in_* as a function of frequency. For sample PPy@H/S/CF the imaginary part of *Z_in_* cancels at 10.6 GHz, while its real part becomes very close to *Z_o_*, so that matching occurs and a minimum of *RL* is achieved. A contrario for sample H/S/CF the imaginary part of impedance never cancels, and the real part is too low, so that matching cannot appear. This is due to the too low values of dielectric constant and loss tangent factors of sample H/S/CF.

**Figure 10:**
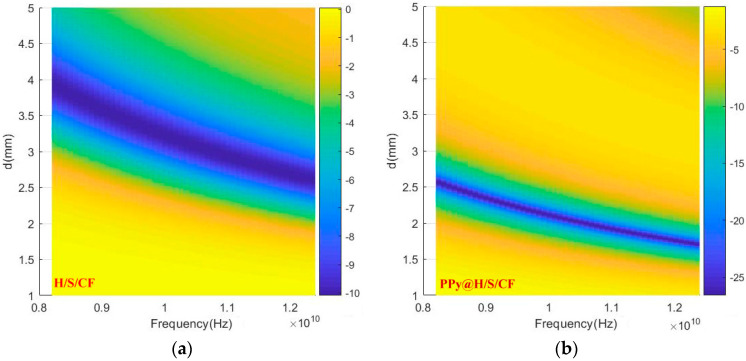
Color map of *RL* as function of frequency and thickness *d*. (**a**): sample H/S/CF, (**b**): sample PPy@H/S/CF.

This excellent value for RL can be tuned over the X band by varying the thickness *d* of the absorber. This is illustrated in Figure 10 showing as color map the intensity of *RL* in dB as a function of frequency and thickness, according to Equation (1). For *d* = 2 mm, minimum *RL* occurs indeed around 10.5 GHz, for higher values of *d*, the frequency is lower, and vice versa. While for sample H/S/CF, *RL* remain globally higher than −10 dB for all values of *d* and frequency.

**Figure 11:**
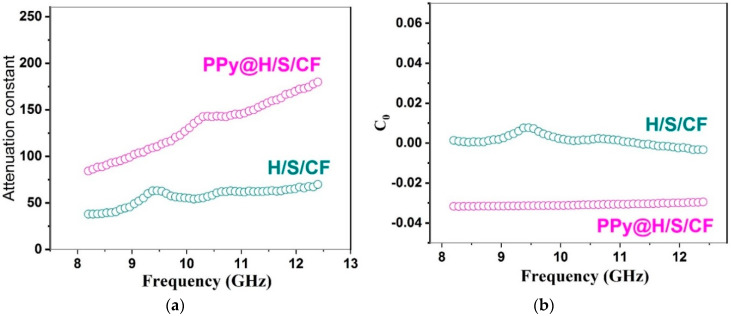
(**a**) Attenuation constant *α* from Equation (3). (**b**) eddy current loss *C*_0_ from Equation (2) in H/S/CF and PPy@H/S/CF samples.

Finally Figure 11 shows the attenuation constant *α* = *Re* (*γ*) from (1c) provided by each composite. As expected, PPy@H/S/CF sample provides superior attenuation in accordance to dielectric and magnetic losses observed in Figure 7.

The eddy current loss coefficient *C*_0_ and microwave attenuation constant *α* are expressed by the following equations:

(4) $$C_{0}=\mu^{\prime \prime }{\mu^{\prime }}^{-2}f^{-1}=2\pi \mu_{0}\sigma d^{2}$$

(5) $$\alpha =\frac{2\pi }{c}\sqrt{2\mu^{\prime }\varepsilon^{\prime }}\sqrt{\frac{\mu^{\prime \prime }\varepsilon^{\prime \prime }}{\mu^{\prime }\varepsilon^{\prime }}-1+\sqrt{{\left(\frac{\mu^{\prime \prime }}{\mu^{\prime }}\right)}^{2}+{\left(\frac{\varepsilon^{\prime \prime }}{\varepsilon^{\prime }}\right)}^{2}+{\left(\frac{\mu^{\prime \prime }\varepsilon^{\prime \prime }}{\mu^{\prime }\varepsilon^{\prime }}\right)}^{2}+1}}$$

where *d*, *µ*_0_, and *σ* represent thickness, permeability in vacuum and electrical conductivity, respectively. As expected from Equation (5), which shows that *α* is proportional to *ε*″ and *μ*″, so that the attenuation constant is higher for Py@H/S/CF than for H/S/CF sample, this is owing to the respective values shown in Figure 7. As known to all, if the magnetic loss is caused by the eddy current loss mechanism, then *C*_0_ values should be constant by increasing the frequency [31]. This is indeed verified in Figure 11b.

**Figure 12:**
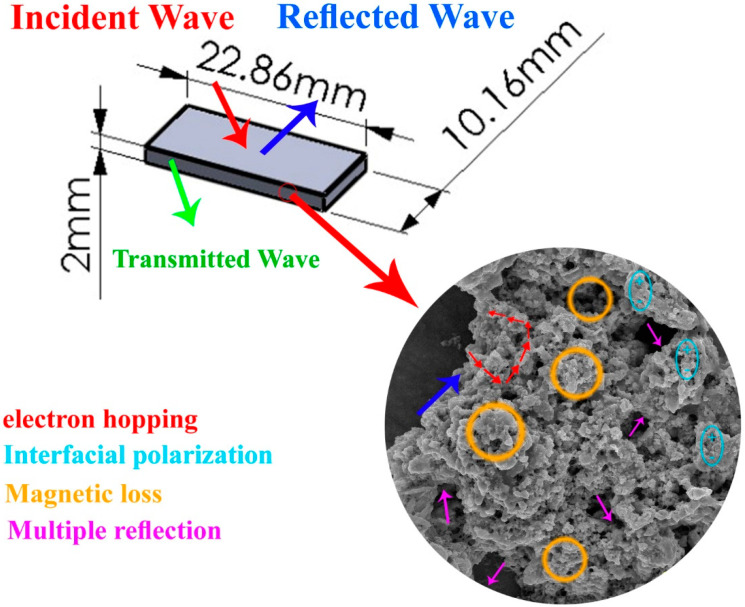
Mechanisms involved in reflection and attenuated transmission in our samples.

As concluding comment, the different mechanisms assumed to be involved in reflection and attenuated transmission in our samples are illustrated in Figure 12.

## 4. Conclusions

In this paper, we presented a microwave absorber composite based on a Salisbury screen configuration using as an absorbing material CoFe_2_O_4_/NiFe_2_O_4_/Carbon fiber (H/S/CF) composite coated with polypyrrole polymer (PPy@H/S/CF) through sol-gel and in-situ polymerization processes. Structural and morphological analysis were performed. TEM images show that CFs are surrounded and decorated well with submicron-sized magnetic particles and H/S particles are almost tightly agglomerated on the surface of carbon fiber. The microwave measurements reveal the superior performances of the PPy@H/S/CF sample compared to the H/S/CF sample and literature since it exhibits a return loss *RL* = −55 d B and a −10 dB bandwidth of 5 GHz.
